# The value of combined detailed first-trimester ultrasound–biochemical analysis for screening fetal aneuploidy in the era of non-invasive prenatal testing

**DOI:** 10.1007/s00404-023-07267-3

**Published:** 2023-11-08

**Authors:** Caixia Ye, Hongyan Duan, Mengyuan Liu, Jianqiang Liu, Jingwen Xiang, Yizhen Yin, Qiong Zhou, Dan Yang, Ruiling Yan, Ruiman Li

**Affiliations:** The First Affiliated Hospital of Jinan University, Guangzhou, 510630 China

**Keywords:** First-trimester screening, Major trisomies, Down syndrome, Ultrasound screening, Non-invasive prenatal testing

## Abstract

**Purpose:**

This study aimed to investigate the performance, cost-effectiveness and additional findings of combined detailed ultrasound and biochemical screening for risks of major fetal trisomies in the first-trimester.

**Methods:**

This is a retrospective analysis study, we estimated the risk of trisomies 21, 18 and 13 based on maternal age, fetal nuchal translucency thickness, nasal bone, ductus venosus pulsatility index velocity, tricuspid regurgitation, fetal heart rate, free beta-human chorionic gonadotropin, and pregnancy-associated plasma protein A in singleton pregnant women, and performed non-invasive prenatal testing for women with risks of trisomy 21 between 1:500 and 1:300. Invasive diagnostic testing was performed for women with positive or failed non-invasive prenatal testing result and in the high-risk group of this screening method. The direct costs were compared between this strategy and the non-invasive prenatal testing which alone used as first-line screening for all pregnant women.

**Results:**

Among 25,155 singleton pregnant women who underwent screening, 24,361 were available for analysis, of these, 194 cases underwent non-invasive prenatal testing. Among the 24,361 women, 39, 19, and 7 had trisomies 21, 18 and 13, respectively. The use of this strategy could potentially detect approximately 94.87% of trisomy 21 cases, 100% of trisomy 18 cases, and 100% of trisomy 13 cases, with false-positive rates of 2.49%, 0.41%, and 0.49%, respectively. The overall detection rate and overall false-positive rates were 96.92% and 2.52%, respectively. The detection rate was 100% in the advanced age group and 94.12% in the general age group. Additionally, structural abnormalities were detected in 137 fetuses, and 44 fetuses had other chromosomal abnormalities. The total cost of this strategy was $3,730,843.30, and the cost per person tested was $153.15. The total cost of using non-invasive prenatal testing as the first-line strategy would be $6,813,387.04 and the cost per person tested was $279.68.

**Conclusions:**

Our strategy is an efficient and cost-effective approach for detecting major trisomies and identifying more fetuses with a potential abnormality. Therefore, this strategy is a valuable screening method and highly feasible in the clinical setting.

## Introduction

Trisomies 21 (T21), 18 (T18), and 13 (T13) are common chromosomal aneuploidies, and T21 is the most common one [1], and its incidence rate is approximately 14.7 per 10,000 population, with an annual increase of 23,000–25,000 cases in China [2]. T21 greatly affects not only growth and development but also the intelligence level of children, imposing a huge mental and economic burden on the family and society. The estimated life- course cost for individuals with Down syndrome in Changsha, China, is $740,811.14 [3].

In China, the most common approach for detecting the risk of major fetal trisomies is a combination of ultrasound assessment, biochemical assessment of maternal serum markers, and maternal age in the first and second trimesters [4]. The karyotype of high-risk cases is clinically determined by amniocentesis or villocentesis. Combined first-trimester screening (CFTS), which uses a combination of serum free β-human chorionic gonadotropin (β-hCG), pregnancy-associated plasma protein-A (PAPP-A), nuchal translucency (NT) thickness, and maternal age to calculate the risk of major fetal trisomies, is the most commonly used approach for prenatal screening for major trisomies in the first trimester [4, 5]. In China, biochemical screening for T21 in the second trimester of pregnancy continues to be widely performed, but its specificity is low, with a sensitivity of 60–70% [6, 7].

In addition to the aforementioned traditional screening methods, non-invasive prenatal testing (NIPT) is preferred by some pregnant women because of its high sensitivity and specificity as well as low false-positive rate (FPR) and false-negative rate (FNR) [8, 9]. Recent research has focused on the economic analysis of the use of NIPT in screening for major trisomies [10–12]. NIPT is highly expensive when chosen as the first-line screening approach for Down’s syndrome. Kagan et al. applied a combination of a detailed ultrasound examination and measurement of NT as the approach for T21 during first-trimester screening, showed a significantly reduced FPR [13]. When NIPT is applied as second-line screening after CFTS, it could reduce the need for invasive testing and the screening costs [14–16].

Currently, China has implemented the three-child policy, a family planning policy implemented by China to actively respond to the aging of the population, the number of Advanced-age and high-risk pregnancies increased [17]. Therefore, screening for major fetal trisomies has become very important, and a screening approach suitable for both advanced- and general-age pregnant women is particularly needed in China. The present study aimed to evaluate the potential performance and cost-effectiveness of combined detailed ultrasound and biochemical screening for risks of T21, T18, and T13 in 11–13^+6^ weeks’ gestation in singleton pregnant women.

## Materials and methods

The present study was performed based on the retrospective data, collecting 25,155 singleton pregnant women who were being screened for aneuploidies at 11–13^+6^ weeks’ gestation (i.e., when the fetal crown-rump length [CRL] was between 45 and 84 mm) at The First Affiliate Hospital of Jinan University from January 2016 to December 2021, which was approved by the Scientific and Ethics Review Committees of the hospital. Notably, the Scientific and Ethics Review Committees had waived informed consent for the study as the nature of the present study is retrospective. The inclusion criteria were as follows: (1) singleton pregnancy, (2) maternal age ≥ 18 years, and (3) CRL measurement ranging from 45 to 84 mm. The exclusion criteria were as follows: (1) vanishing twins and (2) data unavailability of final outcomes (karyotype or children’s health). All pregnancy outcomes were tracked by reviewing obstetrical or neonatal records in our center or by contacting mothers who delivered in other hospitals by telephone approximately six months after delivery to collect details about the fetal chromosomes or the health of the infant. This research study was conducted retrospectively from data obtained for clinical purposes. Our hospital ethics committee has confirmed that no ethical approval is required.

Singleton pregnant women underwent our screening method during their first trimester, and the required data were collected. However, only those pregnant women who had definite follow-up data were included in the analysis, and we obtained the results of fetal karyotype analysis and details of the infants’ health. For the assessment of serum-free β-hCG and PAPP-A were collected at 10–13 gestational weeks, that is, a median of 7 days before ultrasound scanning. Pregnant women were asked to measure their height and weight on the day of the detailed ultrasound examination, declare their ethnicity, mention whether they smoked during pregnancy, report whether they had a history of diabetes and pregnancy, and indicate the method of conception. The required data were collected and inputted into the Astraia software system (Astraia Software GmbH, Munich, Germany). The β-hCG and PAPP-A data were adjusted for multiples of the median (MoM) based on the woman’s height, weight, ethnicity, smoking status, and method of conception by Astraia software system. In the present study, detailed ultrasound examination was performed by doctors in fetal medicine and sonographers, who have undertaken specialized training and/or continuing training and obtained the Certificate of Competency issued by The Fetal Medicine Foundation (FMF; https://fetalmedicine.com/). The first-trimester ultrasound examination and measurement methods of ultrasound parameters adhere to the International Society of Ultrasound in Obstetrics and Gynecology (ISUOG) recommendations [18] and the guidelines of FMF 11–13^+6^ weeks’ scans. The ultrasound parameters were assessed only by experienced sonographers who are certified to examine FMF 11–13 weeks’ scans. In addition to NT thickness, nasal bone (NB), tricuspid regurgitation (TR), ductus venosus pulsatility index velocity (DV-PIV), and fetal heart rate (FHR) measurements, a detailed ultrasound examination was performed.

The ultrasound examination was performed using Voluson E6, E8, E10, and 730 Expert (GE Healthcare). Pregnant women’s ethnicity, age, height and weight, past medical history, pregnancy history, smoking history, ultrasound parameters, β-hCG, and PAPP-A, all of which are routinely used in our centers for first-trimester screening, were recorded, and the data were inputted in Astraia software.

**Fig. 1:**
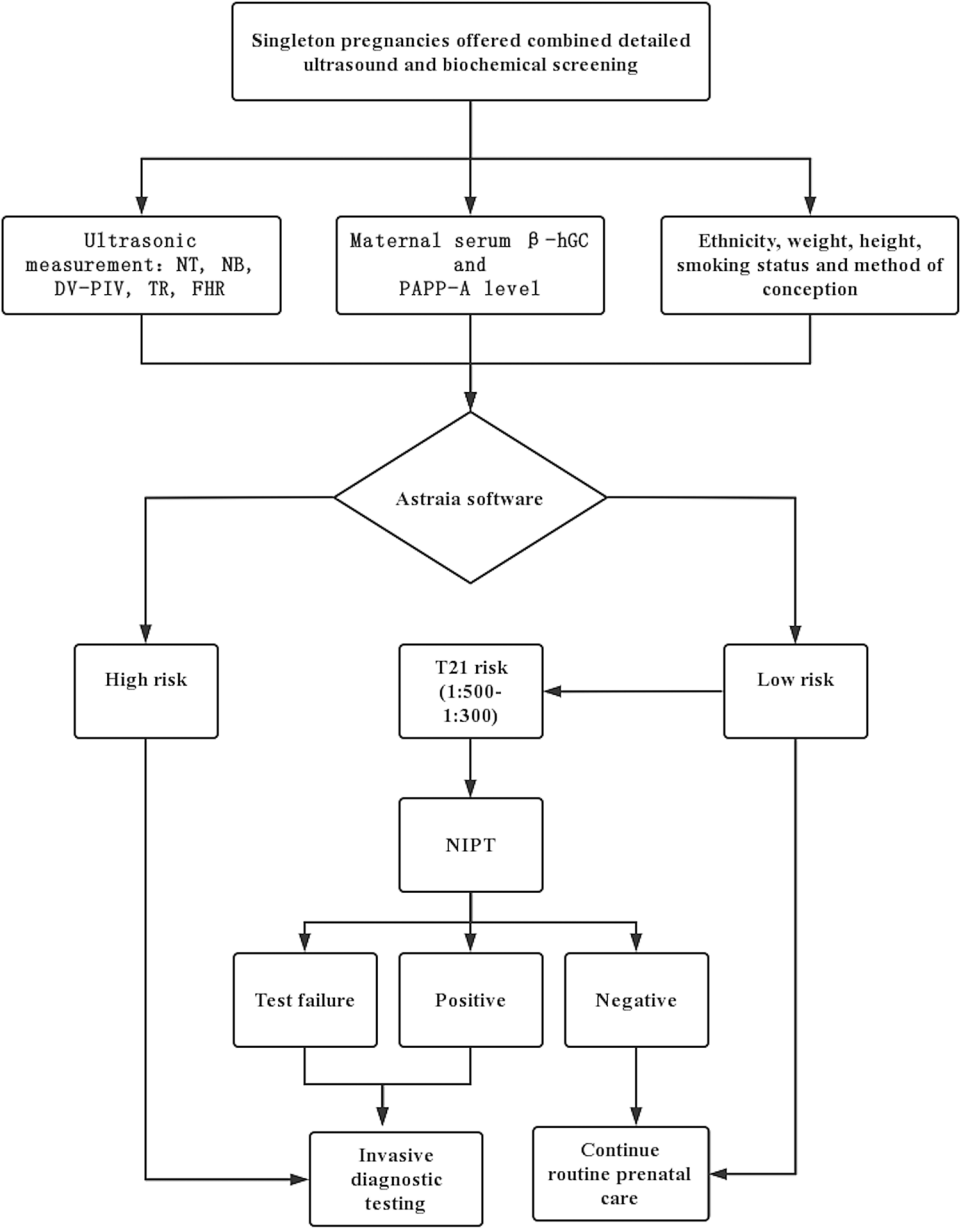
Flowchart of the combined ultrasound and biochemical screening

The risks of major fetal trisomies were calculated using Astraia software by combining maternal age, NT, NB, TR, DV-PIV, FHR, and the MoM of β-hCG and PAPP-A. Women were classified into low-risk (T21 [< 1:300], T18 and T13 [< 1:100]), and high-risk (T21 [> 1:300], T18 and T13 [> 1:100]) groups according to their trisomy risk. These cutoff values are referenced to the FMF. Additionally, NIPT was offered to women with risks of trisomy 21 between 1:500 and 1:300. And invasive diagnostic testing (IDT), such as amniocentesis (18–24 weeks) and chorionic villus sampling (CVS; 11–14 weeks), was offered to women with a positive or failed NIPT result for trisomy and in the high-risk group for fetal karyotyping. Pregnant women with a negative NIPT result and in the low-risk group did not require any further testing (Fig. 1). All participants in the high-risk group and those who had a NIPT-positive and failed result received genetic counseling. All fetuses diagnosed with T21, T18 and T13 were terminated by induction of labor.

In cost analysis, the cost of first-trimester ultrasound scanning, dating ultrasound, maternal serum testing, NIPT, IDT and life-course cost for individuals with Down syndrome was set at $57.95, $28.23, $24.07, $231.80, $304.98 (average cost of amniocentesis [$282.02] and CVS [$327.93]) and $740,811.14, respectively. These charges are based on the price standards of the Guangzhou Price Bureau. The total cost of this strategy, including all costs for first-trimester ultrasound scanning, maternal serum testing, NIPT, IDT, and life-course costs of prenatal missed diagnosis of T21.

We estimated the cost effect of the NIPT strategy when it was used as the first-line approach for all pregnant women and when IDT was performed in high-risk and failed cases. We made an assumption that the detection rate (DR), false-positive rate (FPR), and failure rate of NIPT for the major trisomies were 99.3%, 0.2%, and 1% [11, 19, 20], respectively.

The data of T21, T18, and T13 for the low- and high-risk groups were assessed by statistical analyses using SPSS, version 24.0 (IBM Corp., Armonk, NY, USA). Enumeration data were expressed as rates, and comparisons were made using the chi-square test or Fisher’s exact test. *p* values of < 0.05 were considered to indicate a statistically significant difference. For quantitative variables, two independent groups were compared using the Mann–Whitney test, and for categorical variables, the chi-square test was used.

## Results

**Fig. 2:**
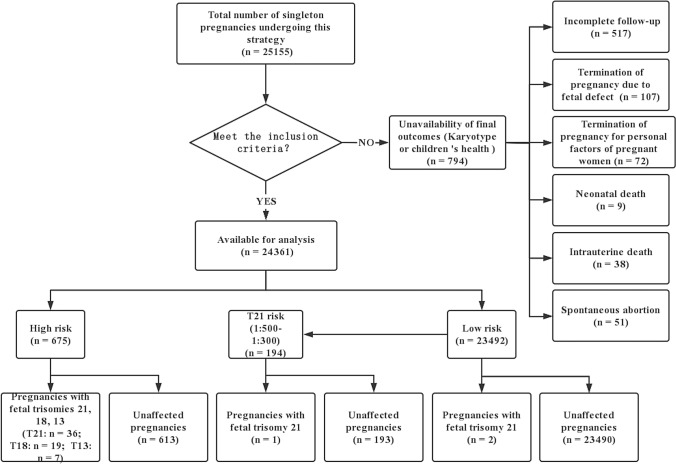
Flowchart of enrollment

In this study, we examined 25,155 singleton pregnant women. Among them, 24,361 met the inclusion criteria and were recruited to the study, and 794 (3.16%) were excluded because data on the fetal chromosome karyotype or infant’s health could not be obtained, which included loss of follow-up (n = 517; 43 cases were at high risk; 13 cases with T 21 risk between 1:500 and 1:300 refuse NIPT and IDT), termination of pregnancy due to fetal defects (n = 107; 50 cases were at high risk), termination of pregnancy due to maternal personal factors (n = 72; 21 cases were at high risk), spontaneous abortion (n = 51; 6 cases were at high risk), intrauterine fetal death (n = 38; 7 cases were at high risk), and neonatal death (n = 9; no case was at high risk). Among the 24,361 women, 643 (2.64%) had a T21 risk of ≥ 1 in 300, and 194 of them were included for NIPT; 119 (0.49%) and 162 (0.66%) women had a T18 and T13 risk of ≥ 1 in 100, respectively; 675 (2.77%) women had a T21 risk of ≥ 1 in 300 or a T18 or T13 risk of ≥ 1 in 100 (Fig. 2). The average age of the enrolled pregnant women was 29.96 ± 4.41 years, and 3,394 (13.93%; 3,394/24,361) women were ≥ 35 years.

Pregnancies that did not have T21, T18, or T13 as assessed in prenatal karyotype analysis or pregnancies that yielded neonates with a normal phenotype were classified in the unaffected group. Moreover, 24,296 of 24,361 fetuses did not have T21, T18, or T13 in the total population, including 24,252 fetuses with a normal fetal karyotype or infant phenotype and 44 fetuses with other chromosomal abnormalities (trisomy 16, n = 1; trisomy 4, n = 1; sex chromosome aneuploidies, n = 6; Turner’s syndrome, n = 15; mosaicisms, deletions, or translocations, n = 21). In the affected group, 65 cases had a prenatal diagnosis of T21 (n = 39), T18 (n = 19), and T13 (n = 7). The details of ultrasound parameters and maternal serum parameters in fetuses with T21, T18, and T13 are shown in Tables 1, 2, and 3, respectively. In case number 6 (a 29-year-old multigravida) and case number 16 (a 33-year-old multigravida) in the low-risk group, amniocentesis was performed because of the absence of an NB and the patient’s worry. All cases in the affected group led to the termination of pregnancy or intrauterine fetal death. In our strategy, the overall DR of the major trisomies was 96.92%, and the overall FPR was 2.52%; the DR for T21, T18, and T13 were 94.87%, 100%, and 100%, respectively; the corresponding FPR were 2.49%, 0.41%, and 0.64%, respectively. To evaluate the performance of our strategy, we calculated DR, FPR, positive predictive value (PPV), and negative predictive value (NPV) based on the results of chorion or amniocyte karyotyping and the neonatal phenotype (Table 4).

**Table 1:** Details of sonographic makers and maternal serum among fetuses with trisomy 21

No	CRL (mm)	NT (mm)	NB	DV-PIV	TR	FHR (bpm)	Free-beta hGC(MoM)	PAPP-A (MoM)	Risk caculation
1	59.8	**5.8**	**+**	**1.5**	**+**	159	**3.679**	0.709	1/2
2	49.5	**3.6**	−	1.1	**+**	175	1.783	0.990	1/21
3	81.1	**3.5**	−	**1.6**	**+**	157	1.000	1.000	1/2
4	66.7	**4.1**	**+**	**2.3**	−	162	1.602	1.078	1/2
5	53.3	2.3	**+**	1.0	−	164	**2.742**	**0.647**	1/28
6	62.4	1.3	**+**	**1.5**	−	164	1.898	0.731	1/586
7	61.2	**4.4**	**+**	1.2	−	156	**2.454**	0.259	1/2
8	54.7	**5.7**	**+**	**2.1**	**+**	164	**2.678**	1.972	1/2
9	69.0	**5.1**	−	1.1	−	153	1.268	**0.673**	1/183
10	52.9	**6.6**	**+**	**1.4**	**+**	175	**2.270**	**0.660**	1/2
11	52.9	**6.6**	**+**	**1.8**	−	165	1.352	0.410	1/2
12	62.8	**3.0**	−	**1.5**	−	152	**2.378**	**0.554**	1/2
13	76.5	**5.3**	**+**	1.2	**+**	150	**3.188**	2.018	1/2
14	60.4	**3.3**	**+**	**1.8**	−	167	1.287	0.849	1/2
15	59.1	**3.9**	−	1.2	**+**	169	1.146	0.332	1/2
16	53.7	1.5	**+**	1.2	−	167	**2.538**	1.260	1/1010
17	62.6	4.0	−	1.0	−	164	**5.938**	0.364	1/6
18	66.6	**3.3**	**+**	0.9	−	170	1.648	**0.594**	1/3
19	63.5	**8.9**	**+**	1.2	**+**	169	1.498	0.124	1/2
20	59.1	2	−	0.9	**+**	163	0.942	0.169	1/4
21	54.7	**5.9**	**+**	**3.2**	−	**178**	**3.136**	0.286	1/2
22	63.6	**2.8**	−	1.3	−	170	**2.053**	0.772	1/112
23	72.8	**5.9**	**+**	1.3	**+**	159	**2.698**	**0.468**	1/2
24	67.4	**3.5**	**+**	1.2	−	169	1.444	**0.504**	1/2
25	60.4	**5.4**	**+**	**2.4**	**+**	156	1.359	**0.530**	1/2
26	59.8	2.3	**+**	1.1	−	160	1.187	0.262	1/2
27	59.0	2.1	−	1.3	−	170	**3.428**	0.726	1/224
28	56.2	2.4	**+**	1.2	−	176	0.755	0.745	1/80
29	77.0	**4.8**	**+**	1.3	**+**	164	1.005	0.177	1/2
30	61.4	**4.2**	**+**	**2.8**	−	162	**7.062**	0.879	1/2
31	73.1	2.5	**+**	1.1	−	160	1.265	**0.501**	1/25
32	52.8	**5.1**	**+**	**2.5**	−	169	**3.407**	0.871	1/2
33	62.6	**3.0**	**+**	1.3	−	159	**4.063**	0.124	1/2
34	75.4	**2.7**	−	1.3	−	160	**3.441**	1.207	1/431
35	77.3	**13.7**	−	**1.4**	**+**	168	0.191	**0.605**	1/4
36	46.4	**3.0**	**+**	**2.4**	−	174	**2.926**	0.362	1/2
37	62	**2.9**	**+**	1.3	−	156	1.619	0.286	1/2
38	55.5	2.3	**+**	**1.4**	−	171	**2.461**	0.164	1/2
39	63.6	2.3	**+**	**1.4**	−	164	**2.390**	1.606	1/76

+, positive for abnormal findings; −, normal; CRL, crown-rump length; NT, nuchal translucency; NB, nasal bone; DV-PIV, ductus venosus pulsatility index velocity; TR, tricuspid regurgitation; FHR, fetal heart rate

Bold value indicates the item is abnormal

**Table 2:** Details of sonographic makers and maternal serum among fetuses with trisomy 18

No	CRL (mm)	NT (mm)	NB	DV-PIV	TR	FHR (bpm)	Free-beta hGC(MoM)	PAPP-A (MoM)	Risk calculation
1	51.5	2.3	**+**	**2.7**	−	164	0.468	**0.246**	1/6
2	56.5	2.1	−	1.0	**+**	158	**0.299**	**0.163**	1/13
3	49.1	**5.6**	−	**3.2**	−	161	**0.058**	**0.193**	1/2
4	46.5	**7.8**	**+**	**+**	−	**179**	**0.115**	**0.146**	1/2
5	60.7	**10.4**	−	**1.4**	**+**	166	0.348	**0.211**	1/2
6	83.2	**2.9**	**+**	**1.5**	−	151	1.491	**0.205**	1/10
7	60.7	**5.9**	−	1.1	−	**142**	0.353	**0.186**	1/2
8	51.2	1.7	**+**	**1.4**	−	174	**0.182**	**0.074**	1/6
9	68.7	**11.5**	−	0.9	−	164	0.506	**0.263**	1/2
10	51.8	**4.8**	−	**1.4**	−	**181**	0.384	**0.191**	1/10
11	48.6	**7.0**	**+**	**2.4**	**+**	162	0.434	**0.219**	1/2
12	55.0	**3.1**	**+**	**3.3**	**+**	168	**0.236**	0.325	1/2
13	66.3	**3.9**	−	1.3	−	**147**	**0.065**	**0.069**	1/2
14	59.6	**4.0**	**+**	1.1	−	155	0.329	**0.166**	1/2
15	63.4	1.9	−	**3.1**	−	173	0.346	**0.068**	1/3
16	69.0	1.6	−	1.2	−	155	0.312	**0.166**	1/96
17	63.1	**6.2**	**+**	**2.5**	−	172	**0.032**	**0.090**	1/2
18	57.2	**7.8**	**+**	**2.2**	−	157	0.520	**0.208**	1/2
19	65.9	**10.11**	**+**	1.3	**+**	172	0.503	0.390	1/4

**+**, positive for abnormal findings; −, normal; CRL, crown-rump length; NT, nuchal translucency; NB, nasal bone; DV-PIV, ductus venosus pulsatility index velocity; TR, tricuspid regurgitation; FHR, fetal heart rate

Bold value indicates the item is abnormal

**Table 3:** Details of sonographic makers and maternal serum among fetuses with trisomy 13

No	CRL (mm)	NT (mm)	NB	DV-PIV	TR	FHR (bpm)	Free-beta hGC(MoM)	PAPP-A (MoM)	Risk calculation
1	53.9	2.2	**+**	**1.5**	−	**182**	**0.319**	**0.420**	1/2
2	78.8	**9.4**	−	**2.2**	−	**190**	**0.28**	0.875	1/3
3	55.5	**5.3**	**+**	1.2	−	**182**	0.732	**0.546**	1/2
4	65.1	2.5	**+**	1.2	−	**189**	1.541	1.02	1/44
5	61.0	**4.7**	**+**	**1.7**	−	**201**	0.517	**0.467**	1/2
6	67.4	2.1	−	1.0	−	**174**	0.743	0.209	1/4
7	54.7	1.7	−	1.3	−	**188**	**0.426**	**0.507**	1/24

+, positive for abnormal findings; −, normal; CRL, crown-rump length; NT, nuchal translucency; NB, nasal bone; DV-PIV, ductus venosus pulsatility index velocity; TR, tricuspid regurgitation; FHR, fetal heart rate

Bold value indicates the item is abnormal

**Table 4:** Performance of first-trimester CUB with NIPT for T21, T18 and T13

Trisomy	Affected		Unaffected		PPV (%)	NPV (%)
	Positive/total	DR (%)	Positive/total	FPR (%)		
21	37/39	94.87	607/24322	2.49	5.75	99.99
18	19/19	100.00	100/24342	0.41	15.97	100.00
13	7/7	100.00	155/24354	0.64	4.32	100.00
21, 18, 13	63/65	96.92	613/24296	2.52	9.32	99.99

DR, detection rate, also known as true positive rate; FPR, false positive rate; PPV, positive predictive value; NPV, Negative predictive value

In this study, 3,394 women were of advanced age, that is, ≥ 35 years. Among them, 234 (6.89%, 234/3394) were at high risk of T21, T18, or T13 and 31 had a final diagnosis of T21, T18, or T13. Moreover, 20,967 women were younger than 35 years, with 442 (2.11%, 442/20,967) being at high risk (including one case of moderate risk for NIPT failure), and 34 had a final diagnosis of T21, T18, or T13 (including 2 cases of low risk). The DR was 100% in the advanced age group and 94.12% in the general age group. No significant difference was noted in the DR of T21, T18, or T13 between different age groups (*P* = 0.864).

During the first trimester, all singleton pregnant women were examined in a detailed manner by ultrasound. Among the 25,155 pregnant women, 137 had detection of fetal structural abnormalities, including 50 cases of fetal heart defects (confirmed by ultrasound again after 16–18 weeks), 18 cases of upper lip cleft or palate, 16 cases of multiple malformations, 14 cases of fetal hydrops, 11 cases of holoprosencephaly, 7 cases of omphalocele or gastroschisis, 6 cases of megalocystis, 5 cases of anencephaly or exencephaly, 5 cases of fetal limb abnormality, 3 cases of absent ductus venosus, 1 case of encephalocele, 1 case of abnormal pedicle syndrome, 1 case of amniotic band syndrome, and 1 case of spina bifida. Of the 137 women with fetal structural abnormalities, 52 underwent amniocentesis or choriocentesis for prenatal diagnosis, and 18 cases involved chromosomal abnormalities; 85 women refused to undergo prenatal diagnosis and chose labor induction directly, including 52 (61.2%, 52/85) cases in the high-risk group of aneuploidy, 33 (38.8%, 33/85) cases in the low-risk group.

In this strategy, all 24,361 pregnant women underwent the first-trimester ultrasound examination, with 194 undergoing NIPT and 676 undergoing IDT (Fig. 2). The total cost when using this strategy was $3,730,843.30, and the cost of testing per person was $153.15 (Table 5). If all 24,361 singleton pregnant women received dating ultrasound and NIPT, then the total cost would be $6,813,387.04, and the cost of testing per person would be $279.68 (Table 5).

**Table 5:** The costs of screening major trisomies with CUB + NIPT and NIPT strategy

	Ultrasound		Biomarker	NIPT	IDT	Misdiagnosis	Cost	
	Scaning ($57.95)	Dating ($28.23)	($24.07)	($231.78)	($304.98)	($740,811.14)	Total	Per person
CUB + NIPT	2,4361	0	2,4361	194	676	2	$3,730,843.30	$153.15
NIPT	0	2,4361	0	2,4361	357^a^	0.5^b^	$6,813,387.04	$279.68

NIPT, non-invasive prenatal testing; IDT, invasive diagnostic testing; CUB, combined ultrasound and biochemical

^a^357 = 65 + (24361 − 65) × 0.2% + 24361 × 1% (assuming 0.2% of FPR and 1% of failure rate)

^b^0.5 = 65 × (1 − 99.3%) (assuming 99.3% of DR)

## Discussion

### Main findings of the study

The use of NIPT for prenatal screening for common trisomies could reduce the number of IDT in pregnant women [21, 22], and it may help avoid the loss of a fetus resulting from IDT prenatally [23, 24]. Furthermore, the application of NIPT has shown substantially better outcomes over traditional methods, which could increase the DR of affected fetuses and reduce the number of affected births in high-risk and general populations [25]. However, NIPT is relatively expensive and is not suitable as a first-line screening method for chromosomal aneuploidy [26]. In China, NIPT is currently mainly used for women of the intermediate-risk group of traditional screening method, women who have contraindications to IDT prenatally, and pregnant women who miss the best time for serological screening.

The present study demonstrated that this screening method for major fetal trisomies, achieving a DR of 96.92% and an FPR of 2.52%, which is superior to traditional screening methods [27, 28]. Using NIPT in pregnant women with T 21 risk between 1:500 and 1:300 that has shown an improved DR and a reduced FPR, thus reducing the number of patient counseling sessions and the need for IDT prenatally. Among the 24,361 women, 39 (0.16%) had T21, 19 (0.08%) had T18, and 7 (0.03%) had T13, and the incidence of T21 was similar to that reported previously [2]. With our strategy, the FNR was 5.13% for T21, which is much lower than those obtained with traditional screening methods (21.21%, 27.27%, 30.77%, and 30% by CFTS, quadruple screening, triple screening, and double screening, respectively) [27]. Additionally, no false-negative cases of T18 and T13 were found. The present study demonstrated that our strategy, when compared with traditional screening methods, may reduce the birth rate of fetuses with major trisomies to achieve the goal of prepotency.

In this study, women of advanced maternal age accounted for 13.93% (3,394/24,361) of the total pregnant women. However, the number of confirmed cases of T21, T18, or T13 in this group accounted for 47.69% (31/65) of the total confirmed cases of T21, T18, or T13 in this study. This suggests that the risk of major fetal trisomies in pregnant women of advanced maternal age is significantly higher than that in pregnant women from the general population, with no significant difference in the DR among the two pregnant women age cohorts. This finding indicates that our strategy for screening T21, T18, or T13 in the first trimester is suitable for pregnant women of all ages.

As reported in our previous study [29], a detailed ultrasound examination of the fetus might be effective in identifying some chromosomal abnormality during the first trimester and in diagnosing a significant proportion of severe structural deformity. A recent study [30] showed that the DR of fetal structural abnormalities through first-trimester ultrasound scans was 43.1%, including abnormalities in the nervous system, abdominal wall, major cardiac system, and genitourinary system, along with any facial, limb, and skeletal malformations. Recently updated guidelines have also emphasized the importance of detailed anatomic evaluation of the fetus in the first trimester [31]. In this study, the fetal structure was scanned for a detailed evaluation while measuring related ultrasound parameters. Among all singleton pregnancies, 137 fetuses had structural abnormalities (0.54%, 137/25,155). Detailed ultrasound examination of the fetus during the first trimester can help detect a significant proportion of fetal structural defects at an early stage [13]. This approach allows pregnancies involving fetuses with fatal defects to be terminated at an early stage, thus reducing, to a certain extent, the physical and psychological impact and economic losses to pregnant women. Moreover, the examination of the genetic material of fetuses with structural defects is helpful in discovering genetic diseases, which is conducive to the genetic counseling of future pregnancy and promoting eugenics.

### Comparison with previous studies

The CFTS, quadruple screening, triplescreening, and double screening is a common traditional method for screening major fetal trisomies in China. Among the four traditional screening methods, CFTS is the most commonly used one. Luo et al. conducted a retrospective analysis of the four traditional methods together with NIPT in terms of screening performance and cost-effectiveness, and the results showed that CFTS had the highest DR when used as a first-line screening method (93.94%) and had cost benefits [27]. With a contingent strategy based on conventional screening, together with NIPT for intermediate-risk cases, the DR for all major trisomies was 88.9%, and the FPR was 1.3% [32]. In another study, NIPT contingent on results from CFTS showed DRs of 91.5%, 100%, and 100% for T21, T18, and T13, respectively [33].

Kagan et al. used a combination of maternal age, NT thickness, and DV-PIV, together with NIPT, and reported DRs of approximately 96.3%, 94.9%, and 90.7% for T21, T18, and T13, respectively, at an FPR of 0.85% [34]. In their study, at the current NIPT cost, this strategy was more cost-effective than NIPT for screening all pregnant women. However, the cost calculations in the above study did not consider the financial loss of the surviving missed cases, whereas in the our study, the calculated cost included an economic loss from the birth of missed cases, and the data were collected from real-world cases, not a modeled analysis.

In our strategy, 0.80% (194/24,361) of women were offered NIPT. NIPT was cost-effective when used as a second-line approach for screening major fetal trisomies, as demonstrated previously [35].

### Clinical implications

If NIPT is used as a first-line screening strategy, assuming a 99.3% DR, 0.2% FPR, and 1% failure rate [11, 20, 36–38] and if all women with positive and failed cases receive IDT prenatally and genetic counseling, then the cost will be $279.68 to screen a pregnant woman, whereas, with our strategy, the cost of screening per case will be only $153.15 (Table 5). At present, in Mainland China, only pregnant women under 35 years old with intermediate-risk of aneuploidy in early pregnancy serological screening or high-risk pregnant women with amniocentesis contraindication are eligible to apply for free NIPT. Early pregnancy serological aneuploidy screening and ultrasonography are free for pregnant women with maternity insurance.

NIPT is not recommended as a first-line screening method because of the current cost analyzed [26]. Currently, NIPT remains expensive, particularly in developing countries such as China. Even among advanced-age pregnant women, at the current price of NIPT ($222.88–$356.61 per woman in China), the cost of NIPT as a first-line method for screening Down’s syndrome is too high to be suitable for the first-line application, but for very old pregnant women (≥ 39 years old), NIPT as a first-line screening strategy is cost-effective [39]. In developing countries, this high-performance testing [40], be used for part of pregnant women rather than all, can not only save cost but also decrease the number of IDTs prenatally while increasing the DR.

By contrast, NIPT is limited to screening for the risk of genetic material, with a focus on T21, T18, T13, and sex chromosome abnormalities, and it has a certain failure DR and cannot detect structural abnormalities of the fetus [26, 41]. With our strategy, a total of 137 fetuses with malformations were detected, which provides important evidence for early genetic diagnosis and more time for couples to take counseling and make decisions accordingly. This screening method also improves the DR of other chromosomal abnormalities that cannot be screened by NIPT alone. With our strategy, 1 case of trisomy 16; 1 case of trisomy 4; and 21 cases of chromosome mosaicism, deletion, or translocation were detected.

All pregnant women in our hospital will be screened for fetal structural abnormalities in the second trimester of pregnancy. Two false-negative cases were detected in our study: case number 6 and case number 16. In both cases, the diagnosis of T21 was made through amniocentesis because of the absence of NB after ultrasonography of fetal structure in the second trimester, resulting in the final termination of the pregnancy.

Our strategy improved the DR of these major trisomies and reduced its FPR. Additionally, to some extent, our approach helped some pregnant women to avoid IDT prenatally. At the same time, the DR of other chromosomal abnormalities and fetal structural abnormalities also increased.

### Research implications

This study is of great significance for the screening of fetal aneuploidy and fetal structural abnormalities in the first trimester. This method can also be used to predict the risk of preeclampsia and fetal growth restriction by adding the measurement of uterine artery pulse index to the ultrasound examination. In the future, the predicted risk of preeclampsia and fetal growth restriction in pregnant women can be followed up and retrospectively analyzed.

### Strengths and limitations

This study had a retrospective and single-center design. In this study, detailed ultrasound examination facilitated the identification of fetal defects in the first trimester, but detailed ultrasound examinations may not be feasible in some regions. However, the detailed ultrasound examination in the first trimester of our study followed the guidelines of FMF 11–13^+6^ weeks’ scans and the ISUOG recommendations [18] and can be, therefore, repeatable.

## Conclusions

The findings of our study confirm that our screening method is not only has high performance but is also cost-effective for screening major fetal trisomies in Chinese populations. This screening strategy is suitable for not only pregnant women of general age but also pregnant women of advanced age. The policy also allows screening out a considerable proportion of fetuses with structural defects in the first trimester to achieve eugenics better and promote the development of eugenics.

## Data Availability

The data that support the findings of this study are available on request from the corresponding author. The data are not publicly available due to privacy or ethical restrictions.
